# Strategies to use tablet computers for collection of electronic patient-reported outcomes

**DOI:** 10.1186/s12955-014-0205-1

**Published:** 2015-01-22

**Authors:** Kara Schick-Makaroff, Anita Molzahn

**Affiliations:** Faculty of Nursing, University of Alberta, Level 3, Edmonton Clinic Health Academy, 11405-87 Ave, Edmonton, AB T6G 1C9 Canada

**Keywords:** Electronic patient-reported outcomes, ePRO, Tablet computers, iPad, Nursing

## Abstract

**Background:**

Mobile devices are increasingly being used for data collection in research. However, many researchers do not have experience in collecting data electronically. Hence, the purpose of this short report was to identify issues that emerged in a study that incorporated electronic capture of patient-reported outcomes in clinical settings, and strategies used to address the issues.

**Findings:**

The issues pertaining to electronic patient-reported outcome data collection were captured qualitatively during a study on use of electronic patient-reported outcomes in two home dialysis units. Fifty-six patients completed three surveys on tablet computers, including the Kidney Disease Quality of Life-36, the Edmonton Symptom Assessment Scale, and a satisfaction measure. Issues that arose throughout the research process were recorded during ethics reviews, implementation process, and data collection. Four core issues emerged including logistics of technology, security, institutional and financial support, and electronic design.

**Conclusions:**

Although use of mobile devices for data collection has many benefits, it also poses new challenges for researchers. Advance consideration of possible issues that emerge in the process, and strategies that can help address these issues, may prevent disruption and enhance validity of findings.

## Introduction

Electronic capture of patient-reported outcomes (ePROs) data [1-4], offers new opportunities to both researchers and clinicians. The benefits and challenges of ePROs previously addressed in the literature [1-16] are summarized in the below list. Although guidelines for reporting patient-reported outcomes have been established [13,17-19], utilization of ePROs is a more recent development. Over the past decade, researchers have described how they worked with electronic data collection systems, platforms or registries of ePROs [3,4,6,11,12,15,16,20]. However, pragmatic issues and potential strategies that a researcher might use when conducting a study to collect ePRO data are not often discussed, and this paper addresses this gap in the literature.

### Benefits and challenges of administering ePROs

#### Benefits

- Immediate access to retrievable data [1,6]
- Potential to link ePRO data with electronic health records [6,12,13]
- Capacity to trigger automated alerts when patients identify problems [1,3,6,11-13]
- Immediate scoring and presentation of data [2,5-7,12]
- More economical in terms of resources and time [3,4,6-8,12,15]
- Greater likelihood of reporting sensitive information [1,2,5]
- Prevention of errors in data-entry [1,4]
- Observation of survey completion in real-time [1,5,6,11]
- Improved data quality [3,4]
- Less missing information [1,2,8,12]
- With minor modifications from paper to electronic formats, validation is not likely necessary [1,6,7,9]
- Patient satisfaction with electronic platform [1,2,5,7,11,14]
- Storage of longitudinal data [2,12]
- Web-based platforms allow reporting from multiple locations (ie home, clinic etc.) [2,6,12,15,16]
- Enhanced communication and effectiveness of multidisciplinary care [3,6,11]
- ePRO data can be collected independently from encounters with healthcare providers [6,11]
- Educational materials for patients can be tailored to their scores [5,6,11]

#### Challenges

- Greater expense and upfront planning compared to paper administration [1,6,12]
- Necessity of training of clinical staff, researchers or patients [1,6]
- Resistance in a healthcare culture to electronic administration [1]
- Inadequate information technology structures in research sites (ie computers or mobile devices, personnel, secure servers) [1,12]
- Instruction and supervision may be required by people with limited computer experience [12,14]
- May encounter challenges linking ePRO data to patient health records or other datasets [3,5,6]
- Integrating ePRO data into clinic workflow and interpretation of clinical relevance [6]

### Purpose and research question

The purpose of this research was to identify issues that emerged in a study that incorporated ePROs in clinical settings and to identify possible solutions/strategies to address the issues.

## Methods

The overall focus of the pilot project was the feasibility of using tablet computers to collect ePROs in two outpatient dialysis clinics [14]. People attending a clinic, in one of two cities in Western Canada, were invited to participate if they were over 19 years old, did not have mild-severe cognitive challenges, could speak and read English, and were medically stable. Fifty-six dialysis patients volunteered. They were primarily older adults with a mean age of 66 ± 12 (36–91 years old), and only two had used a tablet computer previously. Ethics approval was attained from a University Human Research Ethics Board and a health authority. While in the clinic waiting room, participants completed two ePROs (Kidney Disease Quality of Life-36 ((KDQOL-36)) [21]), and Edmonton Symptom Assessment Scale ((ESAS)) [22]) and a patient satisfaction measure. The tablet computer used in this study was the iPad™ (iPad is a trademark of Apple, Inc). The ESAS was designed as a new application (app), and the KDQOL-36 was completed through *KDQOL Complete* [23] providing online scores in real time. The ESAS app had minor modifications, and measurement equivalence was addressed through cognitive debriefing and usability testing [9,14].

Interpretive description, an established qualitative methodology used in applied clinical fields, is an inductive approach that offers ways to consider themes/patterns from subjective descriptions, and create descriptions that inform clinical understanding [24]. It was used to identify the core ePRO issues that arose and strategies that were used during the design and conduct of the project. The researchers qualitatively recorded issues that arose during ethics reviews and data collection. Researchers used field notes and emails to each other to formally document the issues. Notes were updated when issues were resolved and new matters arose [24,25]. Analysis was undertaken at a conceptual/thematic descriptive level, identifying thematic patterns developed *in situ* from the data [25]. If another researcher wanted to examine and verify our decision-making and interpretation of findings in the interests of rigor, audit trails (documentation and reflexive writing) were created throughout the analytic process.

## Results

The themes that emerged relating to issues and strategies to address ePRO implementation included: logistics of technology, security, support, and design. Table 1 provides excerpts from field notes to support these themes.

**Table 1:** **Findings and checklist**

**Theme**	**Excerpt from field notes to support thematic findings**	**Checklist for ePRO use in clinical settings**
Logistics of technology	Started brainstorming ideas to deal with how I am attending to infection control: sanitizing screen? Using stylus? Hand sanitizer before hand (and give to participant as a gift)? Disposable screen covers? Others? I also want to talk [electronic provider] to see if they have other suggestions.	Have you attended to infection control?
	Found out today that [health authority] has not formulated a strategy for patient wireless access and requires staff members to have individual accountability for their access. As a researcher, I am neither “patient” nor “staff”. [Information Technology] confirmed that I cannot access their internet. Alternative option is to use 3G [3rd generation of mobile telephone/cellular technology].	Do you have access to the internet in the hospital/clinic/health authority?
	Advised to test iPad’s 3G connections in both home dialysis clinics. Hospital building structures and medical equipment could interfere with 3G signal.	Have you tested the tablet in the clinical setting?
	We decided to set up a new contract for one of the iPads with [a different cellular provider] because in [2nd city], they have a tower nearby. Patients from [2nd city] have told me that they have better reception with this provider.	
	Using 3G will obviously have implications for printing – cannot print from iPad without internet. I need to contact [computer programmer] about purchasing router to connect iPad to printer - but without using internet signal.	Do you need to print from the tablets?
	The renal services are in the process of moving from paper to electronic charting. But they aren’t there yet. In consultation with the nurses, they said they want results printed and given to them on paper.	Do you need to access or synch with electronic health records?
Security	[Privacy analyst] told me I need a minimum of 8 characters for the passwords on the iPads. Need to change all passwords on all iPads accordingly.	Do you have your tablets password protected and encrypted?
	[Health authority] wants to know if devices will be dedicated to research use, or if they will be brought in as-needed. Definitely dedicated for research use only – will follow up with [research assistants] when they sign confidentiality agreements too.	
	Although the iPads will be securely stored in my locked office when they aren’t in use in the hospitals, we need to also have them securely stored on the days they are used for data collection. In consultation with [research assistant], we are going to buy a small box that locks and is on wheels (for easy transport of equipment and files). We’ll keep the iPads locked in the box when a patient is not using them.	Where will you securely store your tablets?
	To meet [health authority] ethics standards, we need to back up all data on Health Research Data Repository [HRDR; at University of Alberta] at the end of every day of data collection.	Where are you storing your data?
	Must receive confirmation of data transfer from HRDR *before* deleting data off of iPads.	Are you frequently backing up your data?
Support	[Health authority] has a requirement, as mandated by the Ministry of Health and the OIPC [Office of the Information and Privacy Commissioner], to encrypt all mobile devices used in their facilities. Need to provide proof that the encryption is enabled on tablets.	Do you have support/approval from the Information Management/Information Technology department in the health setting?
	[The nurse clinician] agreed that the ward clerk can ask patients, when she calls them to remind them of their appointment, that if they are planning to participate in the study, they need to come 15 minutes early – which is fabulous! If patients are late, they will not be invited to participate. Data collection is *so* different when it is happening “in real time” in a clinic setting.	
	Requested approval to buy another iPad. With 3 research assistants in the clinic each day, there are times when they all could be using one with a patient. As it is now, sometimes they are waiting for a tablet, and more than anything else, we can’t interrupt the flow of the clinic.	Do you have financial and technological support for tablet development?
	Need to start to set up invoice with KDQOL Complete prior to data collection so I can trial data entry onsite.	
Design	Met with [academic professor] who is using iPads with healthcare professionals. Even though they are assuming many participants will know how to use tablets, they are still opting to go with a very simple layout with drop-down menu bars. The simpler, the better.	Do you need to adapt the screen display for a specific population?
	Read an article on computer use with seniors. They recommended using large font, black writing on white/plain background, and no distracting images on the screens. So definitely no pop-ups or flashing stars when a survey is completed.	
	Met with [computer programmer] and he is using FileMaker Go to design the app. We’ll meet at each phase to test and revise the design. And when we are done, he’s going to provide a step-by-step set of instructions (with pictures) for use of the app, download, and transfers.	Are you creating new apps to collect research data?
		If yes, are you using data input time stamps?
	Time stamps will be date/minute/second. In this way, they can also be used as a back-up, secondary participant identifier because I’ll know the order of participants who volunteered on any given day at the home dialysis clinics.	
	Coons et al. [9] – need to undertake cognitive debriefing and usability testing to assess clarity, understanding, and usability of the technology. I think I would like to include brief interviews with each participant after they use the iPad.	Have you attended to measurement equivalence of the electronic versus paper formats?

The *logistics for use of the technology* in the clinical environment required considerable time and attention. In our study, the health authority did not allow researchers to access the healthcare institution’s intranet, and because one survey was conducted online, tablets needed to have 3G (cellular) capacity. Given that health settings often have variable cellular receptivity, the tablets needed to be tested in various clinical locations. In this project, it was not possible to synchronize ePRO and electronic health data because the clinics were using a hybrid of paper and electronic records. Logistical issues arose due to the need for wireless printing. Because clinicians requested that ePRO results be printed for their use in real time, printing options required advance onsite testing.

Health administrators, practitioners, and patients all asked about how infection control was being managed with the tablet. We used two approaches. First, participants were asked to use a hand sanitizer. Second, everyone used a stylus to prevent touching the screen. Older adults who had not used a touch-screen tablet previously found the stylus very helpful.

*Technology security* was paramount, and it came under scrutiny during ethics reviews. While KDQOL-36 and ESAS data was printed for clinicians, raw data was downloaded daily to the university data repository so that no surveys were stored on the tablet. As a result, a file transfer protocol was required.

KDQOL-36 data was stored on the *KDQOL Complete* database, housed on a US server. Under the US Patriot Act, the US Government could access this data without knowledge/consent by researchers or participants. To uphold confidentiality, ID codes were used to identify participants and this was explained in the informed consent forms.

To ensure tablets were securely stored, they were kept in a locked cabinet in the researcher’s office when not being used. When being used, they were either with the researcher or kept in a locked storage container. To meet university/health authority ethical requirements, tablet computers needed 8-digit passwords and the highest levels of encryption.

*Institutional and financial support* was essential. Ethical review for the pilot project took approximately one year, primarily due to the novelty of the technology in the clinical setting. Approval was needed from the health authority’s Information Technology department to ensure that their security requirements were met and health privacy legislation was followed. Financial support was necessary to assist with purchasing tablets and printers, hiring computer programmers, procuring online survey support, and accessing cellular internet.

*Elements of electronic design* were issues that emerged in relation to the screen display, creation of new apps, and measurement equivalence. Because participants were primarily older adults without computer experience, screen displays that were easily readable were needed. We kept the app minimalistic with large font, black writing on white background, and no distracting graphics. Time stamps were also used to log data input as an audit trail. Given that both the ESAS and KDQOL-36 were originally designed for paper, there was a need for only minor modification of the instruments for the tablet [9].

## Discussion

This study offers strategies that researchers/practitioners might consider useful when employing ePROs, a topic rarely discussed in the literature. The findings are presented in Table 1 along with a checklist of questions that researchers may ask themselves when using ePROs in clinical settings.

Given that every clinical application of ePROs will be unique, different issues may emerge in diverse contexts, and testing in real-world conditions is imperative [4]. For example, technology logistics in clinical environments may dictate the capacity to which ePRO data may be linked to electronic health records. In light of the current expansion of organizations incorporating electronic patient records, combined with the desire for immediate retrieval of electronic data, the potential to integrate ePROs with electronic records will only continue to expand [1,6,12,13]. Strategies for such integration will be paramount as researchers strive for utility of ePROs in clinical practice.

Specific clinical settings may also direct logistics controlling the spread of infection. Strategies other than those used in this study could include sanitizing the tablet screen between each use or using disposable screen covers.

Institutional requirements regarding technology security will vary based on national/local policies and contexts. Specifically, passwords and encryption requirements will differ in various healthcare settings. Documentation of security features is an important consideration, especially during research ethics applications. Only two authors [4,12] mentioned security requirements in the ePRO literature. However, in our project, this was a significant consideration, one that required collaborative strategies with computer programmers/privacy analysts.

Findings from this study regarding institutional and financial support are substantiated in the literature. While some authors [1,6,12] acknowledge that there will be greater upfront costs and training for patients/professionals to use technology, electronic formats are still more economical both in terms of time and resources [3,4,6-8,12,15]. ePROs also offer an environmental, paperless option. Despite international movement towards use of ePROs for the purposes of creating retrievable data [1,6], linking with electronic records [6,12,13], and triggering automated patient alerts [1,3,6,11-13], institutional support was not recognized in the ePRO literature as a critical strategy with the exception of acknowledging support from clinical leaders [4,6]. Research addressing how ePRO data is used as a feedback mechanism to clinicians is an area in need of future development.

Health data privacy requirements will vary based on the legislation, policy, culture or health authority. Establishing early collaborations with local Information Technology departments may assist researchers to overcome challenges with institutional support.

The aspect of electronic design was rarely discussed, with a few exceptions [6,15]. Our findings emphasize the need to tailor design for the intended population. Diverse patient groups may have different technology comfort levels, and design will need to be adapted accordingly. One area of design that has been developed in the literature pertains to modification of measurements for electronic delivery [9]. The level of modification needed for translation from paper to electronic version will determine the evidence needed to test measurement equivalence.

A limitation of this study is that the primary purpose of the overarching project related to the feasibility of ePROs in clinical settings [14] and not on issues that emerged in the use of the technology; thus, systematic questionnaires were not used to identify such issues. Nevertheless, this paper is a form of knowledge translation offered to researchers/clinicians who will use ePROs in future work. This study was only conducted in one health authority and in one country, limiting the breadth of issues encountered.

Although use of mobile devices, specifically tablet computers, for data collection has many benefits, it also poses new challenges for researchers. Advance consideration of possible issues that emerge in the process, and strategies to address these issues, may prevent disruption and enhance validity of findings.

## Abbreviations

App: Application
ePRO: Electronic patient-reported outcome(s)
ESAS: Edmonton Symptom Assessment Scale
KDQOL-36: Kidney Disease Quality of Life
